# Nuclear medicine in the assessment and prevention of cancer therapy-related cardiotoxicity: prospects and proposal of use by the European Association of Nuclear Medicine (EANM)

**DOI:** 10.1007/s00259-022-05991-7

**Published:** 2022-11-05

**Authors:** Matthias Totzeck, Nicolas Aide, Johann Bauersachs, Jan Bucerius, Panagiotis Georgoulias, Ken Herrmann, Fabien Hyafil, Jolanta Kunikowska, Mark Lubberink, Carmela Nappi, Tienush Rassaf, Antti Saraste, Roberto Sciagra, Riemer H. J. A. Slart, Hein Verberne, Christoph Rischpler

**Affiliations:** 1 Department of Cardiology and Vascular Medicine, West German Heart and Vascular Center Essen, University Hospital Essen, University Duisburg-Essen, Essen, Germany; 2 Nuclear Medicine Department, University Hospital, Caen, France; 3 Department of Cardiology and Angiology, Hannover Medical School, Hannover, Germany; 4 Department of Nuclear Medicine, University Medicine Göttingen, Georg-August-University Göttingen, Göttingen, Germany; 5 Department of Nuclear Medicine, Faculty of Medicine, University of Thessaly, University Hospital of Larissa, Larissa, Greece; 6 Clinic for Nuclear Medicine, University Hospital Essen, University of Duisburg-Essen, Essen, Germany; 7 Department of Nuclear Medicine, DMU IMAGINA, Georges-Pompidou European Hospital, Assistance-Publique – Hôpitaux de Paris, University of Paris, Paris, France; 8 Nuclear Medicine Department, Medical University of Warsaw, Warsaw, Poland; 9 Medical Physics, Uppsala University Hospital, Uppsala, Sweden; 10 Department of Advanced Biomedical Sciences, University of Naples “Federico II”, Naples, Italy; 11 Heart Center, Turku University Hospital and University of Turku, Turku, Finland; 12 Nuclear Medicine Unit, Department of Experimental and Clinical Biomedical Sciences “Mario Serio”, University of Florence, Florence, Italy; 13 Medical Imaging Center, Department of Nuclear Medicine and Molecular Imaging, University of Groningen, University Medical Center Groningen, Groningen, The Netherlands; 14 Department of Biomedical Photonic Imaging, Faculty of Science and Technology, Enschede, The Netherlands; 15 Department of Radiology and Nuclear Medicine, Amsterdam UMC, Location AMC, University of Amsterdam, Amsterdam, The Netherlands

**Keywords:** Nuclear medicine, Cancer therapy-related cardiotoxicity, Cardio-oncology, PET, SPECT, Chemotherapy, Radiotherapy, Immunotherapy

## Abstract

Cardiotoxicity may present as (pulmonary) hypertension, acute and chronic coronary syndromes, venous thromboembolism, cardiomyopathies/heart failure, arrhythmia, valvular heart disease, peripheral arterial disease, and myocarditis. Many of these disease entities can be diagnosed by established cardiovascular diagnostic pathways. Nuclear medicine, however, has proven promising in the diagnosis of cardiomyopathies/heart failure, and peri- and myocarditis as well as arterial inflammation. This article first outlines the spectrum of cardiotoxic cancer therapies and the potential side effects. This will be complemented by the definition of cardiotoxicity using non-nuclear cardiovascular imaging (echocardiography, CMR) and biomarkers. Available nuclear imaging techniques are then presented and specific suggestions are made for their application and potential role in the diagnosis of cardiotoxicity.

## Preamble

The European Association of Nuclear Medicine (EANM) is a professional non-profit medical association that facilitates communication worldwide among individuals pursuing clinical and research excellence in nuclear medicine. The EANM was founded in 1985. This position paper is intended to assist practitioners in providing appropriate nuclear medicine care for patients. They are not inflexible rules or requirements of practice and are not intended, nor should they be used, to establish a legal standard of care. The ultimate judgment regarding the propriety of any specific procedure or course of action must be made by medical professionals taking into account the unique circumstances of each case. Thus, there is no implication that an approach differing from this position paper, standing alone, is below the standard of care. To the contrary, a conscientious practitioner may responsibly adopt a course of action different from that set out in the position paper when, in the reasonable judgment of the practitioner, such course of action is indicated by the condition of the patient, limitations of available resources, or advances in knowledge or technology subsequent to publication of the position paper. The practice of medicine involves not only the science but also the art of dealing with the prevention, diagnosis, alleviation, and treatment of disease. The variety and complexity of human conditions make it impossible to always reach the most appropriate diagnosis or to predict with certainty a particular response to treatment. Therefore, it should be recognized that adherence to this position paper will not ensure an accurate diagnosis or a successful outcome. All that should be expected is that the practitioner will follow a reasonable course of action based on current knowledge, available resources, and the needs of the patient to deliver effective and safe medical care. The sole purpose of this position paper is to assist practitioners in achieving this objective. The aim of this article is to review available imaging modalities in nuclear medicine that have been validated or show promise for the assessment and prevention of cancer therapy-related cardiotoxicity (both systemic treatments and radiotherapy). The background of the respective nuclear imaging techniques will be briefly described, and the existing literature reviewed. For the clinical point of view, eligible patients and responsible therapies that cause cardiotoxicity will be summarized. Furthermore, specific application suggestions for nuclear medicine imaging for assessing cardiotoxicity are given. At this point, we would like to point out that, however, this cannot represent a guideline, since there is still not yet sufficient evidence for some of the nuclear medicine applications discussed and these very promising imaging options still need to be validated further.

## Background

**Fig. 1:**
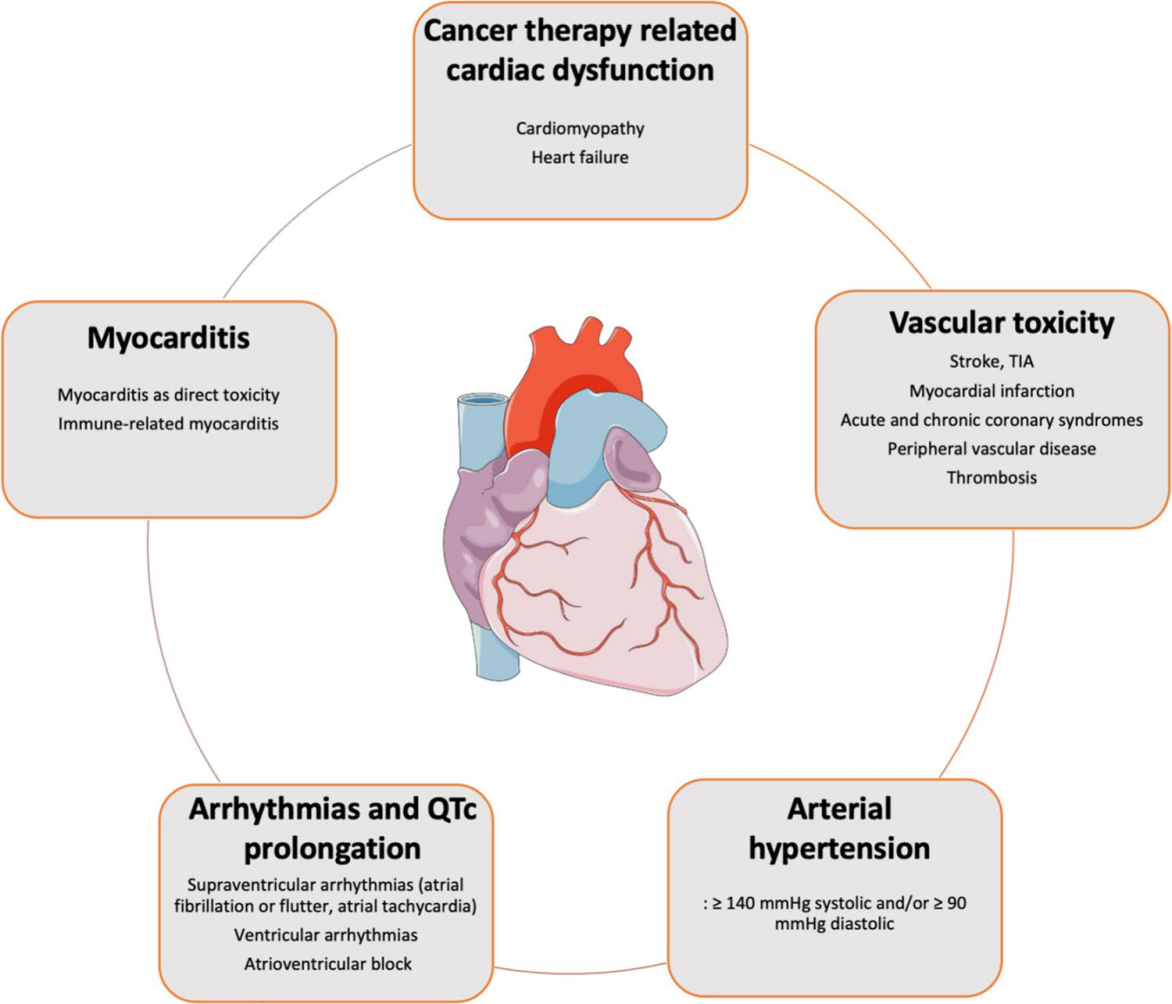
Main cardiovascular toxicities after cancer therapy

Increased survival rates in cancer patients render cardiovascular side effects of cancer therapies increasingly important for long-term morbidity and mortality. Pre-existing cardiovascular diseases, cardiovascular risk factors, genetic predisposition, previous therapies, and growing patient age are associated with an increasing risk for and may aggravate complications following cancer treatments [1, 2]. This ranges from asymptomatic, reversible changes to fulminant, life-threatening complications including heart failure, acute and chronic coronary syndromes, arrhythmias, valvular heart disease, pericardial disease, myocarditis, and thromboembolic complications (Fig. 1). The relatively new field of cardio-oncology aims to identify mechanisms that lead to cardiovascular diseases through cancer and cancer therapy, to establish appropriate diagnostic measures, and to identify the best possible therapy to reduce the burden of cardiovascular disease in cancer patients [1, 2]. The integration of a cardiologist in multidisciplinary cancer care teams to discuss treatment strategies in oncological patients has proven effective in reducing cardiovascular side effects [3]. The establishment of new, targeted cancer therapeutics may generate cardiovascular toxicity and requires close monitoring of patients. Many side effects of novel therapeutics have recently been characterized regarding incidence, mechanisms, and therapeutic approaches [3, 4]. The term cardiotoxicity refers to the various types of damage to the heart or vessels caused by chemical substances, drugs, or ionizing radiation and stem cell transplantation [5]. Table 1 lists various contemporary cancer treatment regimens and their cardiotoxic spectrum.

**Table 1:** Cancer therapies and their main cardiovascular toxicities (modified according to Rassaf et al. [1], Hermann et al. [109], and Rao et al. [110])

Therapy type	Therapy subtypes	Cancer therapy-induced dysfunction	Myocarditis/pericarditis	Arrhythmias/QT prolongation	Vascular toxicity	Hypertension
Conventional chemotherapies	Anthracyclines (doxorubicin, epirubicin)	✓				
	Alkylating agents (cyclophosphamide, melphalan)	✓		✓	✓	
	Antimetabolites (5-FU, capecitabine, cytarabine)	✓	✓cytarabine		✓	
	Microtubule-bonding agents (paclitaxel)			✓	✓	
	Platinum based therapy (cisplatin)				✓	✓
	Antibiotic (bleomycin)				✓	
	Immunomodulatory drugs (thalidomide, lenalidomide, pomalidomide)	✓		✓	✓	
Targeted agents	Proteasome inhibitors (bortezomib, carfilzomib)	✓			✓	✓
	HDAC inhibitors (panobinostat, vorinostat)			✓		
	CDK4/CDK6 inhibitors (ribociclib)			✓		
	mTOR inhibitors (everolimus)	✓		✓	✓	✓
	HER2 inhibitors (pertuzumab, trastuzumab, lapatinib, adotrastuzumabemtansin)	✓				
	VEGF inhibitors (bevacizumab, ramucirumab, aflibercept, sunitinib)	✓		✓bevacizumab	✓	✓
	BCR-ABL1 inhibitors (dasatinib, nilotinib, ponatinib, bosutinib, imatinib)	✓pontinib	✓	✓	✓	✓
	BTK inhibitors (ibrutinib, acalabrutinib, zanubrutinib)			✓	✓ibrutinib	
	ALK inhibitors (alectinib, ceritinib, crizotinib, brigatinib, lorlatinib)		✓ceritinib	✓	✓	✓brigatinib
	BRAF inhibitors (dabrafenib, vemurafenib, encorafenib)	✓		✓	✓encorafenib	✓
	MEK inhibitors (binimetinib, cobimetinib, trametinib)	✓		✓	✓binimetinib	✓
	Multitarget (sorafenib, sunitinib, pazopanib, vandetanib, lenvatinib, regorafenib, cabozantinib)	✓		✓	✓	✓
	EGFR inhibitors (panitumumab, necitumumab)			✓panitumumab	✓necitumumab	✓panitumumab
Immunotherapies	Immune checkpoint inhibitors (ipilimumab, nivolumab, pembrolizumab, atezolizumab, durvalumab, avelumab, cemiplimab, dostarlimab)	✓	✓	✓	✓	
	CAR-T cell therapy (tisagenlecleucel, axicabtagene cioleucel, lisocabtagene maraleucel, brexucabtagene autoleucel, idecabtagene)	✓	✓	✓	✓	
Hormonal therapy	abiraterone, anastrozolem apalutamide, bicalutamide, darolutamide, enzamestane, exemestane, flutamine, letrozole, nilutamide	✓				✓
Radiation therapy		✓	✓	✓	✓	

Arguably, left ventricular dysfunction (cardiomyopathy) and manifest heart failure are the most concerning forms of cardiotoxicity with a profound impact on morbidity and mortality. Heart failure can manifest at an early (e.g., acute or semi-acute) or late phase (e.g., months, years to even decades after potential cardiotoxic treatment). Anthracycline chemotherapy (e.g., doxorubicin, daunorubicin) induces left ventricular dysfunction in a dose-dependent manner [1, 6]. The cardiotoxic-related complication risk is increased by pre-existing cardiovascular risk factors, genetic predisposition, manifest cardiovascular disease, or prior cancer therapy [6]. This cardiotoxic-related complication risk further increases with additional exposure to human epidermal growth factor receptor 2 (HER2) inhibitors, commonly indicated for the treatment of breast cancer [6]. Several other chemotherapeutics are associated with cardiovascular complications: alkylating agents (e.g., cyclophosphamide) can induce severe, early heart failure. Fluoropyrimidines induce endothelial injury that may lead to coronary vasospasms and acute coronary syndromes, with subsequent myocardial ischemia. Various cytotoxic cancer therapies (e.g., cisplatin) in combination with cancer-associated risk factors (i.e., the pro-thrombotic environment associated with cancer) increase arterial and venous thromboembolic complications [6, 7]. The introduction of new, targeted substances has led to new, highly specific forms of cardiovascular toxicity. For example, inhibitors of vascular endothelial growth factor signaling were found to induce arterial hypertension and arterial and venous thromboembolic complications [8]. Various tyrosine kinase inhibitors and serine threonine kinase inhibitors have been associated with left ventricular dysfunction and thromboembolic complications leading to an increased risk of pulmonary embolism [8]. Immune checkpoint inhibitors (ICI) induce severe myocarditis in 1–2% of patients, with a significant risk of major cardiovascular events and mortality [9, 10]. Other complications of the ICI therapies include pericardial disease, acute coronary syndromes, and arrhythmias at higher rates than previously expected [4, 11]. Chimeric antigen receptor (CAR)-T cell therapy is a novel autologous stem cell therapy against hematologic and solid tumors. In the acute phase following transplantation, a severe inflammatory syndrome named cytokine release syndrome (CRS) may occur. CRS but also CAR-T cell therapy may have negative effects on the cardiovascular system leading to acute heart failure and hypotension. This requires further in-depth analysis [5].

As radiotherapy is associated with significant cardiovascular complications, it is important to realize that approximately 35% of cancer patients undergo radiotherapy within 1 year after diagnosis [1]. Radiotherapy-related myocarditis is a known acute complication, but its incidence has decreased due to dose fractioning. Long-term complications of radiotherapy involving the heart (e.g., patients with mediastinal lymphoma, left-sided breast cancer, and lung cancer) include cardiac fibrosis and acceleration of coronary atherosclerosis, and can arise with a latency of several decades after exposure to radiation [12]. These long-term complications include coronary artery disease, valvular disease, and diastolic dysfunction. Radiation during childhood and concomitant exposure to anthracyclines is associated with a significant increased risk for cardiac complications [13].

## Cardiac assessment of patients with signs suspicious of cardiotoxic origin

**Fig. 2:**
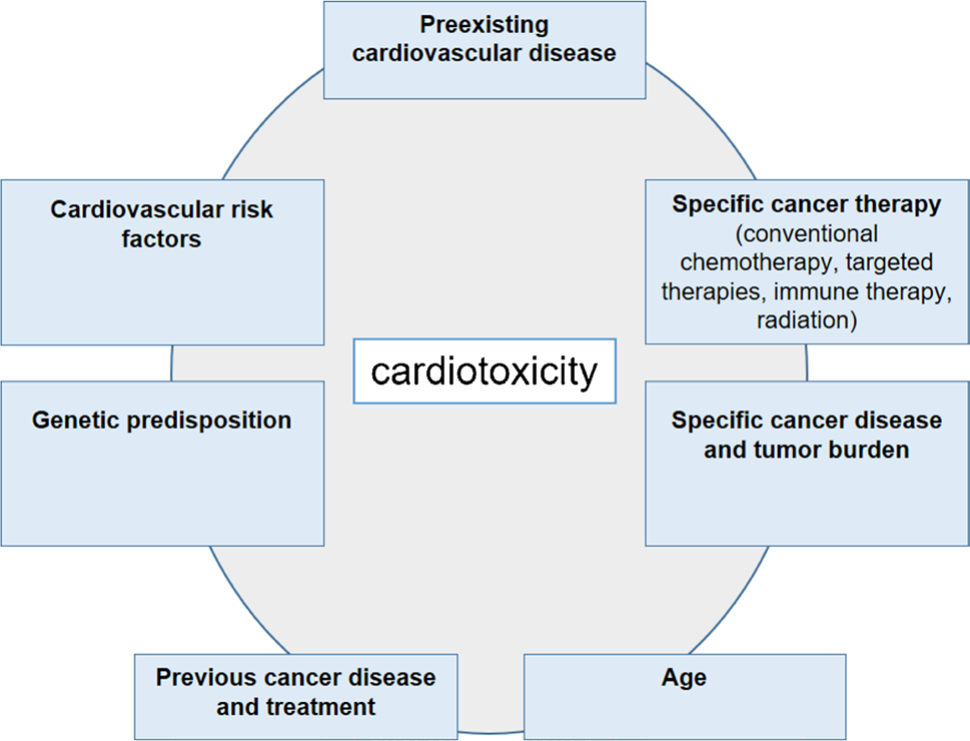
Multiple factors may contribute to an increased risk for cardiotoxicity

Patients with a predisposition for cardiovascular complications from cancer therapy require standardized cardio-oncological monitoring for an early identification and subsequent treatment of cancer therapy-related side effects [1]. Figure 2 shows the multitude of risk factors for the development of cardiotoxicity during the course of cancer and cancer treatment. The diagnostic workflow includes baseline risk assessment prior to therapy [14], monitoring for acute complications during therapy, and long-term follow-up for late cardiovascular side effects [6]. Recent position papers have therefore provided very specific recommendations for individual patients with a predisposition for cardiovascular complications and distinct cancer therapies.

**Fig. 3:**
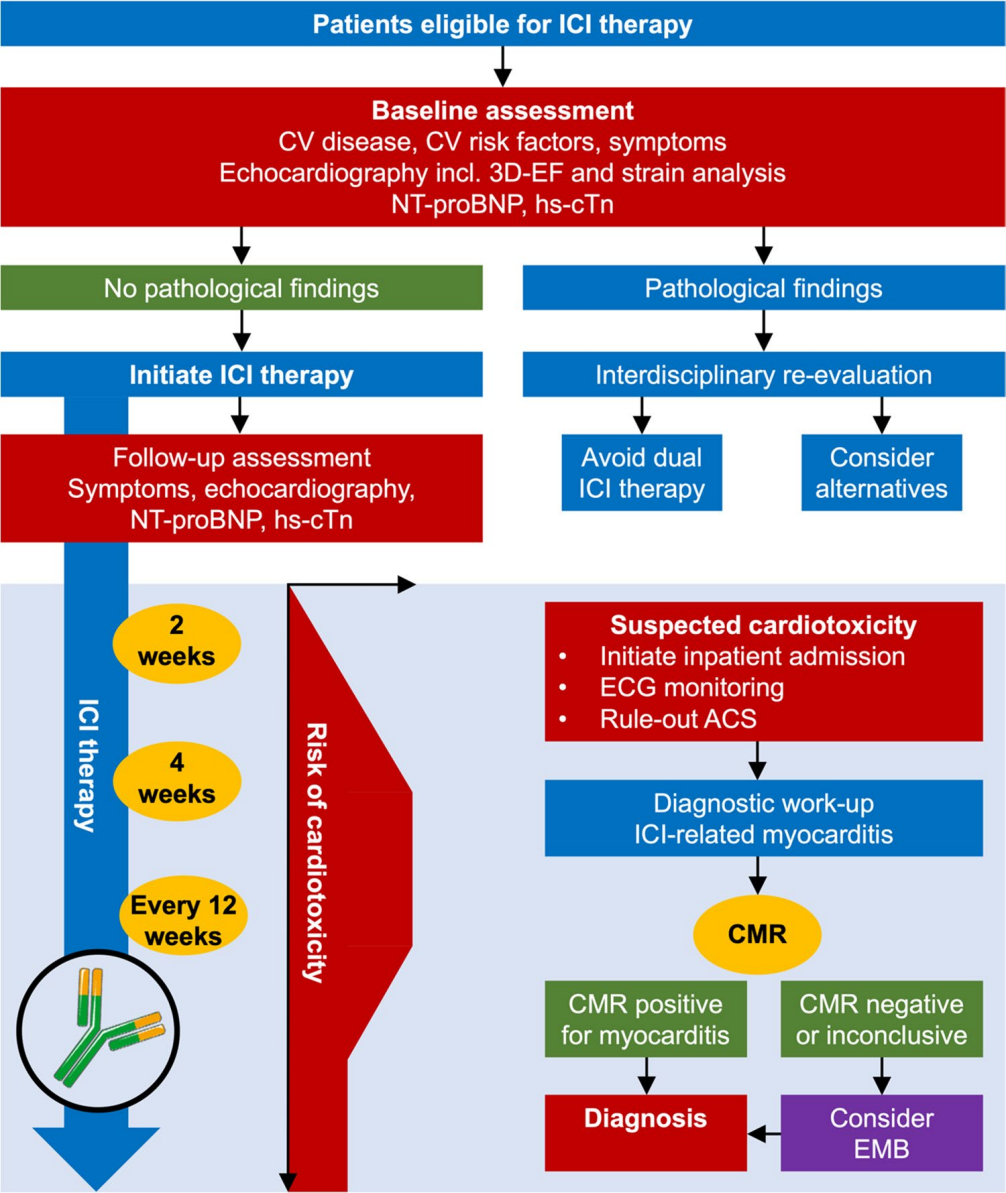
Workflow for the surveillance of patients receiving immune checkpoint inhibitor therapy

At baseline, the assessment of patients requiring cancer therapy includes the assessment of conventional cardiovascular risk factors and pre-existing cardiovascular disease that may aggravate the risk for cardiovascular toxicities [2, 14]. Pre-therapy assessment includes physical examination, electrocardiogram (ECG), cardiac biomarkers (high-sensitive troponin and N-terminal pro-brain natriuretic peptide (NT-proBNP)), and echocardiography depending on the type of therapy. The cardiovascular risk profile of the planned cancer therapy (including conventional chemotherapy, targeted therapies, immunotherapy, or radiotherapy) is evaluated in the context of the patient’s medical history and clinical findings, and may trigger intensified cardio-oncology monitoring or modification of cancer therapy in patients at high-risk to develop cardiac disease of cardiotoxic origin (particularly in those with pre-existing cardiovascular risk factors and disease). A possible, concrete approach to monitoring using nuclear imaging is discussed in the section “Approach for nuclear imaging assessment in the field of cardiotoxicity.” A proposed workflow for the surveillance of patients receiving immune checkpoint inhibitor therapy is outlined in Fig. 3.

Cardio-oncological surveillance during therapy depends on the individual patient’s risk and on the applied therapy. A general cardio-oncology visit includes a clinical examination, ECG, cardiac biomarkers, and echocardiography [6]. Of note, echocardiography is the imaging of choice for the surveillance of cancer patients when ultrasound windows are adequate. Modern, targeted therapies may require close monitoring of particular complications, e.g., QTc prolongation, atrial fibrillation, arterial hypertension, or myocarditis. In patients with suspected cardiotoxicity, further diagnostic modalities can be initiated, e.g., cardiac magnetic resonance imaging (CMR), nuclear imaging technologies, or cardiac catheterization [3, 15]. Echocardiography should include an assessment of left ventricular ejection fraction (LVEF), examination of diastolic dysfunction, and strain analysis. These measures in conjunction with cardiac biomarkers allow grading severity of cancer therapy-related cardiac dysfunction (CTRCD) into “mild,” “moderate,” and “severe” (e.g., mild CTRCD is defined as an LVEF ≥ 50% AND new relative decline in global longitudinal strain (GLS) by > 15% from baseline AND/OR new rise in cardiac biomarkers (troponin I/T > 99th percentile, BNP ≥ 35 pg/ml, NT-proBNP ≥ 125 pg/ml, Table 2) [16]. Novel parameters, e.g., for an advanced assessment of right ventricular function, are currently evaluated in clinical trials [17, 18].

**Table 2:** Definition of cardiovascular toxicities of cancer therapies according to the International Cardio-Oncology Society (IC-OS) consensus statement (according to Herrmann et al. [16])

Cardiotoxicity type	Category	Severity	Diagnosis criteria
**Cancer therapy-related cardiac dysfunction (CTRCD)**	**Asymptomatic** (with or without additional biomarkers, LVEF values are based on 2D echocardiography)	Mild	LVEF ≥ 50% AND new relative decline in GLS by > 15% from baseline AND/OR new rise in cardiac biomarkers (troponin I/T > 99th percentile, BNP ≥ 35 pg/ml, NT-proBNP ≥ 125 pg/ml)
		Moderate	New LVEF reduction by ≥ 10 percentage points to an LVEF of 40–49% New LVEF reduction by < 10 percentage points to an LVEF of 40–49% AND new relative decline in GLS by > 15% from baseline AND/OR new rise in cardiac biomarkers
		Severe	New LVEF reduction to < 40%
	**Symptomatic** (with LVEF and supported diagnostic biomarkers)	Mild	Mild HF symptoms, no intensification of therapy required
		Moderate	Need for outpatient intensification of diuretic and HF therapy
		Severe	HF hospitalization
		Very severe	Requiring inotropic support, mechanical circulatory support, or consideration for transplantation
**Myocarditis**	**Pathohistological diagnosis** Multifocal inflammatory cell infiltrates with overt cardiomyocyte loss by light microscopy of cardiac tissue samples or **Clinical diagnosis** A troponin elevation with 1 major criterion or a troponin elevation with 2 minor criteria after exclusion of acute coronary syndrome or acute infectious myocarditis based on clinical suspicion	Severe	Hemodynamic instability, heart failure requiring non-invasive or invasive ventilation, complete or high-grade heart block, and/or no significant ventricular arrhythmia
		Non-severe (clinically significant)	Symptomatic but hemodynamically and electrically stable, may have reduced LVEF, no features of severe disease
	**Major criterion** CMR diagnostic for acute myocarditis (modified Lake Louise criteria) **Minor criteria** •Clinical syndrome •Ventricular arrhythmia and/or new conduction system disease •Decline in cardiac function •Other immune-related adverse events •Suggestive CMR	Smoldering (subclinical)	Incidentally diagnosed myocarditis without any clinical signs or symptoms
		Steroid refractory	Non-resolving or worsening myocarditis (clinical worsening or persistent troponin elevation after exclusion of other etiologies) despite high-dose methylprednisolone
**Vascular toxicity**	**Asymptomatic**	Atherosclerosis	Coronary artery disease: new corona artery stenosis > 50% on coronary computed tomography angiogram or > 70% on coronary angiogram, or newly abnormal electrocardiogram (ECG), nuclear or echo stress test Peripheral arterial disease: new ankle-brachial index (ABI) value ≤ 0.9 is considered abnormal, with 0.7–0.9 being mildly reduced, 0.4–0.69 moderately reduced, and < 0.4 severely reduced or chance in ABI from baseline by − 0.15 Carotid artery disease: new intima-media thickness (IMT) > 0.9 mm or new plaque on carotid ultrasound, or change in IMT > 0.04/year from baseline
		Thrombosis	Venous thrombosis: new characteristic features and Duplex ultrasound, contrast CT, or venogram Arterial thrombosis: new characteristic features on ultrasound or angiogram, or optical coherence tomography
		Abnormal vasoreactivity	Peripheral: new flow-mediated dilation of the brachial artery (FMD) < 7.1% or reactive hyperemia index (RHI) < 2 on Endo-PAT, or change in FMD or RHI by > 50% from baseline Coronary epicardial: new coronary vasoconstriction (reduction in coronary artery diameter) in response to acetylcholine infusion Coronary microvascular: new < 50% increase in coronary blood flow in response to acetylcholine infusion, or a coronary flow reserve < 2 in response to adenosine
	**Symptomatic**	Stroke	2018 AHA/ASA Guidelines for the Early Management of Patients With Ischemic Stroke An Updated Definition of Stroke for the 21st Century Stroke
		Transient ischemic attack	
		Myocardial infarction	4th Universal Definition of MI
		Acute coronary syndromes	2013 ACCF/AHA Guideline for the Management of ST-Elevation Myocardial Infarction 2014 AHA/ACC Guideline for the Management of Patients with Non–ST-Elevation Acute Coronary Syndromes 2015 ESC Guidelines for the management of acute coronary syndromes in patients presenting without persistent ST-segment elevation 2017 ESC Guidelines for the management of acute myocardial infarction in patients presenting with ST-segment elevation
		Chronic coronary syndromes	2019 ESC Guidelines for the diagnosis and management of chronic coronary syndromes: the Task Force for the diagnosis and management of chronic coronary syndromes of the European Society of Cardiology (ESC)
		Peripheral arterial disease	2017 ESC Guidelines on the Diagnosis and Treatment of Peripheral Arterial Diseases, in collaboration with the European Society for Vascular Surgery (ESVS)
		Vasospastic angina	2019 ESC Guidelines for the diagnosis and management of chronic coronary syndromes: the Task Force for the diagnosis and management of chronic coronary syndromes of the European Society of Cardiology (ESC) International standardization of diagnostic criteria for vasospastic angina
		Microvascular angina	2019 ESC Guidelines for the diagnosis and management of chronic coronary syndromes: the Task Force for the diagnosis and management of chronic coronary syndromes of the European Society of Cardiology (ESC) International standardization of diagnostic criteria for microvascular angina
		Raynaud’s phenomenon	Meeting the diagnostic criteria of an international consensus panel of recurrent episodes bilateral blanching or tricolor change of the fingers
**Hypertension**	Normal SBP ≤ 130 mmHg And DBP ≤ 80 mmHg	Treatment threshold for hypertension before, during, and off therapy/cancer survivors	CVD or ASCVD risk ≥ 10%: ≥ 130 mmHg systolic and/or ≥ 80 mmHg diastolic Otherwise: ≥ 140 mmHg systolic and/or ≥ 90 mmHg diastolic
		Cancer therapy holding threshold	≥ 180 mmHg systolic and/or ≥ 110 mmHg diastolic
		Exaggerated hypertensive response	Systolic BP increase > 20 mmHg or mean arterial BP increase > 15 mmHg
		Hypertensive emergency response	Very high BP elevations associated with acute hypertension-mediated organ damage (heart, retina, brain, kidneys, and large arteries), therefore requiring immediate BP reduction to limit extension or promote regression of target organ damage
**QT prolongation and arrhythmias**	**QTc prolongation**	QTcF < 480 ms	Acceptable: continue current treatment
		QTcF 480–500 ms	Prolonging: proceed with caution; minimize other QT-prolonging medications, replete electrolytes
		QTcF > 500 ms	Prolonged: stop treatment and evaluate. May require dose reduction or alternative therapy
	**Arrhythmias**	Ventricular arrhythmia	2015 ESC Guidelines for the management of patients with ventricular arrhythmias and the prevention of sudden cardiac death 2017 AHA/ACC/HRS Guideline for Management of Patients With Ventricular Arrhythmias and the Prevention of Sudden Cardiac Death
		Ventricular tachycardia (VT), including polymorphic VT (torsades de pointes)	
		Ventricular fibrillation	
		Atrial fibrillation	2020 ESC Guidelines for Management of Atrial Fibrillation 2014 AHA/ACC/HRS Guideline for the Management of Patients with Atrial Fibrillation
		Atrial flutter	
		Atrial tachycardia	2019 ESC Guidelines on Supraventricular Tachycardia 2015 ACC/AHA/HRS Guideline for the Management of Adult Patients With Supraventricular Tachycardia: A Report of the American College of Cardiology/American Heart Association Task Force on Clinical Practice Guidelines and the Heart Rhythm Society
		Supraventricular tachycardia	
		Sinus tachycardia	
		Sinus bradycardia	2018 ACC/AHA/HRS Guideline on the evaluation and management of patients with bradycardia and cardiac conduction delay
		Sick sinus syndrome	
		Atrioventricular block first, second and third degree	
		Conduction disorder (disease)	

*ACC* American College of Cardiology, *AHA* American Heart Association, *ASCVD* atherosclerotic cardiovascular disease, *ASE* American Society of Echocardiography, *BP* blood pressure, *CMR* cardiac magnetic resonance, *CTRCD* cancer therapeutics-related cardiac dysfunction, *DBP* diastolic blood pressure, *GLS* global longitudinal strain, *HF* heart failure, *LVEF* left ventricular ejection fraction, *QTcF* QT interval corrected by the Fridericia formula, *SBP* systolic blood pressure

Moreover, other imaging modalities including CMR have been also proposed for the investigation of cancer therapy-related cardiotoxicity [19]. For anthracycline therapy, the European Society of Medical Oncologists (ESMO) has proposed a guideline which incorporates high-sensitive troponin serial measurements, and recommended echocardiography as the baseline imaging tool because it is widely available, is inexpensive, and does not require ionizing radiation [20]. Indeed, echocardiography is a widely available, low-cost technique. Nevertheless, it is a highly observer-dependent technique, and the image quality is influenced by the acoustic windows obtained. Therefore, echocardiography may not be able to capture mild changes in LVEF when evaluating cancer therapy-related cardiotoxic effects [21]. However, these may be detected with newer methods such as GLS in combination with cardiac troponins [2].

Given its accuracy and reproducibility in LVEF determination, CMR is considered by the American College of Cardiology/American Heart Association (ACC/AHA) as a method to screen for cardiotoxicity in patients undergoing cancer therapy [21]. Moreover, CMR can depict structural changes in the myocardium, including signs of edema and inflammation, prior to the LV dysfunction [22]. On the other hand, CMR disadvantages include the lower availability, higher cost, adverse reaction to gadolinium contrast agents and contraindications related to kidney failure, and the presence of metal devices (e.g., pacemakers, implantable cardiac defibrillators), as well as the lower tolerance by patients due to longer scanning times and/or claustrophobia. In these cases, nuclear imaging can be considered as a second-line imaging approach in patients with poor echogenicity or image quality [20].

The long-term follow-up of patients after completion of cancer treatment is driven by the baseline risk factors and therapy-related risk factors. Patients receiving chest radiotherapy are at high risk for late complications, particularly new-onset coronary artery disease, and require long-term follow-up [1, 2, 23]. In case of suspected cardiotoxicity, patients need to undergo specific diagnostic tests. In case of suspected coronary artery disease, patients may need additional cardiac imaging, including cardiac catheterization and/or nuclear cardiology techniques [12]. In the following sections, the different imaging approaches in nuclear medicine will be discussed for detecting cardiovascular complications related to cancer therapy in oncological patients particularly regarding its potential advantages over echocardiography and CMR.

## Nuclear imaging of therapy-related cardiotoxicity in oncology patients

There are a number of nuclear medicine imaging modalities to detect cardiotoxic damage. The following paragraphs discuss the most commonly used imaging modalities or those with the highest potential from the authors’ perspective. Table 3 provides an overview of the nuclear medicine methods currently used clinically to investigate the occurrence of cardiotoxicity. The majority of the techniques evolve around the assessment of left ventricular function. Table 4 summarizes the radiation exposure from the radiotracers most commonly used in clinical settings to image cardiotoxicity.

**Table 3:** Overview of clinically used nuclear medicine methods for monitoring for the occurrence of cardiotoxicity

Modality	Parameters	Advantages	Disadvantages
ERNA	-Ventricular volumes -Ejection fractions -Systolic and diastolic function -Wall motion -Phase analysis	-Highly standardized -Excellent reproducibility -Observer independent -Virtually no restrictions regarding patient selection (e.g., obesity, cardiac devices) -Evaluation of both right and left ventricles	-No assessment of myocardial perfusion
MPI (SPECT)	-Left ventricular volumes -Left ventricular ejection fraction -Systolic and diastolic function -Wall motion -Phase analysis -Myocardial perfusion (qualitatively)	-Highly standardized -Good reproducibility -Virtually no restrictions regarding patient selection (e.g., obesity, cardiac devices)	-No assessment of right ventricle
MPI (PET)	-Ventricular volumes -Ejection fractions -Systolic and diastolic function -Wall motion -Myocardial perfusion (quantitatively)	-Highly standardized -Good reproducibility -Quantification (MBF, MFR) -Assessment of epicardial and microvascular disease -Detection of even subtle changes	-Limited availability -Relatively expensive
Innervation imaging (e.g., MIBG SPECT, HED PET)	-Distribution and integrity of sympathetic innervation -Sympathetic tone (tracer washout) -Novel tracers, e.g., receptor density	-Changes in innervation appear before structural or functional changes of the heart	-Limited data
Glucose metabolism (2-[^18^F]FDG PET) of the myocardium	-Regional changes in 2-[^18^F]FDG uptake -Quantitative parameters (SUV)	-Changes appear before structural or functional changes of the heart -Patients often receive the examination anyways for their oncological disease	-Many influential factors on 2-[^18^F]FDG uptake (not specific for cardiotoxicity) -Limited data

*ERNA* equilibrium radionuclide angiography, *2-[*^*18*^*F]FDG* 18-fluoro-deoxyglucose, *HED* [^11^C]C-metahydroxyephedrine, *MBF* myocardial blood flow, *MFR* myocardial flow reserve, *MIBG* [^123^I]-metaiodobenzylguanidine, *MPI* myocardial perfusion imaging, *PET* positron emission tomography, *SPECT* single-photon emission computed tomography

**Table 4:** Radiation exposure from the most commonly used clinical nuclear imaging procedures

Procedure	Radiotracer	Effective dose per MBq	Recommended activity	Resulting effective dose	References
ERNA	[^99m^Tc]Tc-RBCs	0.0047 mSv/MBq	555–1110 MBq	2.6–5.2 mSv	[24]
SPECT MPI	[^99m^Tc]Tc-labeled perfusion tracers	[^99m^Tc]Tc-tetrofosmin (stress and rest): 0.0058 and 0.0063 mSv/MBq, respectively [^99m^Tc]Tc-sestamibi (stress and rest): 0.0066 and 0.0070 mSv/MBq, respectively	Depending on protocol (1-day vs. 2-day protocol, stress only, CZT vs. conventional camera etc.): 150–400 MBq (stress), 180–1200 MBq (rest)	0.9–11.2 mSv	[29, 111]
PET MPI	[^15^O]H_2_O	0.0011 mSv/MBq	2 × 400 MBq (stress/rest)	0.8 mSv	[36, 112]
	^82^Rb	0.001 mSv/MBq	2 × 10 MBq/kg (stress/rest)	1.5 mSv	[36, 112, 113]
	[^13^N]NH_3_	0.0027 mSv/MBq	2 × 400 MBq (stress/rest)	1.8 mSv	[36, 112, 113]
Innervation SPECT	[^123^I]mIBG	0.037 mSv/MBq	111–370 MBq	4.1 – 13.7 mSv	[114]
Viability/inflammation PET	2-[^18^F]FDG	0.019 mSv/MBq	185–555 MBq	3.5–10.5 mSv	[112, 115, 116]
Vascular toxicity PET	2-[^18^F]FDG	0.019 mSv/MBq	3–4 MBq/kg	4–7.5 mSv	[94, 112]

*[*^*99m*^*Tc]Tc-RBCs*, [^99m^Tc]Tc-labeled red blood cells; *[123I]mIBG*, [123I]-metaiodobenzylguanidine; *[*^*18*^*F]FDG*, 2-[^18^F]fluoro-2-deoxy-D-glucose; *ERNA*, equilibrium radionuclide angiography; *MPI*, myocardial perfusion imaging; *PET*, positron emission tomography; *SPECT*, single-photon emission computed tomography

## Two/three-dimensional radionuclide angiography

Nuclear medicine techniques have the advantages of being highly examiner-independent, very reproducible, and feasible in almost all patients regardless of various patient conditions such as constitution or implanted devices which often lead to inconclusive results in other imaging modalities or when echocardiography shows poor quality and/or CMR is e.g. not-tolerated/not available. Equilibrium radionuclide angiography (ERNA) can provide valuable information regarding global and regional ventricular function (at rest and/or during stress), as well as cardiac chamber morphology. In particular, ventricular volumes, EF, ventricular wall motion, and diastolic function can be obtained and phase analysis can be performed. The most often applied technique involves the ^99m^Tc radiolabeling of patient’s red blood cells (RBCs) [24]. For autologous RBC labeling, [^99m^Tc]Tc-erythrocytes represent the most commonly used radiotracer. There are three methods for RBC labeling (in vivo technique, mixed in vivo/in vitro technique, in vitro technique), with different labeling efficiency (60–70%, 90%, >90%, respectively) [24]. ECG-gated blood pool planar acquisition constitutes the routine procedure in clinical practice [24]. Single-photon emission computed tomography equilibrium radionuclide angiography (SPECT-ERNA) can also be performed and allows for the assessment of right ventricular function. Cadmium-zinc-telluride (CZT) cameras allow significant reduction of the acquisition time for SPECT-ERNA, improved spatial resolution, and lower radiation exposure of patients [24, 25].

ERNA is a useful technique for the investigation of therapy-related cardiotoxicity, which represents one of the main indications for the examination. ERNA has an excellent reproducibility (with inter- and intra-observer variability of < 5%) for the quantification of LVEF [26]. Therefore, ERNA is often performed serially in order to detect left ventricular dysfunction as an early sign of cardiotoxicity. In addition to LVEF, SPECT-ERNA also allows quantification of RVEF. To date, there are no conclusive data suggesting an additional benefit of quantifying RVEF compared with LVEF in terms of early detection of cardiotoxicity.

One very good example for the use of ERNA is cardiotoxicity from anthracyclines as e.g. used in breast cancer and hematological diseases. An algorithm for LVEF monitoring using ERNA in these patients has previously been suggested (see Table 5) [27]. Similarly, trastuzumab, a humanized monoclonal antibody, acts against the HER2/neu receptor and is known to cause cardiac dysfunction in some patients [21]. There are several protocols for the evaluation of cardiotoxicity in patients undergoing trastuzumab therapy, incorporating a baseline assessment of LVEF and subsequent serial measurements.

**Table 5:** Guideline for the initiation, monitoring, and discontinuation of anthracycline chemotherapy based on the measurements of the left ventricular ejection fraction (LVEF) using equilibrium radionuclide angiography (ERNA) [27]

Baseline ERNA	Treatment initiation	Serial monitoring (with respect to chemotherapy)	Treatment discontinuation
LVEF ≥ 50%	Yes	•At 250–300 mg/m^2^ dose •At 400 mg/m^2^ dose if high risk* •At 450 mg/m^2^ dose •Prior to each dose > 450 mg/m^2^	If LVEF decreases ≥ 10% (EF units) from baseline and reaches < 50%
LVEF 30–50%	Yes	•Prior to each subsequent dose	If LVEF decreases ≥ 10% (EF units) from baseline, or reaches ≤ 30%
LVEF ≤ 30%	No	–/–	–/–

^*^High-risk features: cyclophosphamide therapy, heart disease, mediastinal radiation, and/or abnormal electrocardiogram

Since cancer therapy-related cardiotoxic effects can be regarded as a life-threatening complication of an effective treatment, it is crucial to identify these patients, in order to manage the complications, or (ideally) intervene at the stage of subclinical toxicity [28]. Despite the wide use of echocardiographic techniques in this field, ERNA can offer valuable information, particularly in patients with borderline LV dysfunction, or a need for precise LVEF quantification.

## Myocardial perfusion imaging

### SPECT

Among different modalities to evaluate LVEF, gated myocardial perfusion imaging (MPI) offers the great advantage to evaluate in a single-session LV function and perfusion [29].

The interest in nuclear imaging procedures, particularly in MPI, to assess and monitor cardiotoxicity or to predict cardiovascular complications in cancer patients has recently increased [30, 31, 32, 33, 34]. The majority of data refers to pre-therapeutic cardiac risk assessment [30, 31] and it has been demonstrated that SPECT MPI provides incremental information over clinical risk factors in predicting increased cardiovascular morbidity and death over a 3-year follow-up period in a population of different cancer types regardless of whether chemotherapy has already been initiated [32]. It has previously been demonstrated [33] that radiotherapy-induced perfusion defects on cardiac SPECT scans initially may appear and persist 3 to 6 years post-radiotherapy in a high percentage of patients even in absence of regional wall motion abnormalities or LV function impairments. Furthermore, more severe MPI abnormalities could be detected in breast cancer patients after postoperative radiotherapy as compared to a control group [34].

As stated above, use of the recently introduced CZT cameras allows the reduction of injected activities [25, 35]. The concordance of data obtained by Anger camera and CZT method with a 70% reduction in injected dose have been recently assessed in a population of oncological patients under monitoring due to potential cardio-toxic chemotherapy [25].

SPECT MPI offers a number of advantages over echocardiography and CMR [21]. SPECT MPI allows to examine virtually every patient regardless of difficult examination conditions (e.g., obesity), presence of metal devices, or other diseases (such as kidney failure). Also, SPECT MPI has a very low intra- and interobserver variability, allows accurate assessment of regional and global wall motion, phase analysis, and ventricular volumes in a single examination without the examination protocol having to be extended and its value is validated by studies involving thousands of patients. Compared to CMR imaging in particular, SPECT MPI has the advantage of lower costs and broader availability. Disadvantages of SPECT MPI are radiation exposure of the patient, limited ability to detect structural changes (e.g., small scars, pericardial effusion, or valvular heart disease), and the inability to investigate the right ventricle. However, as mentioned above, SPECT MPI is a very valuable method if echocardiography or CMR cannot be performed, are not available, or if findings are unclear.

### PET—absolute quantification of myocardial blood flow and flow reserve

Perfusion PET can be used to evaluate both the epicardial vessels and the microvascular circulation [36], the impairment of which seems to be a likely consequence of cancer therapy and in particular radiotherapy [23, 37] and may proceed cardiomyopathy and heart failure.

Direct experiences about the use of perfusion PET in the setting of cardiotoxicity assessment during cancer therapy are very limited. Perfusion PET was mainly used in small patient groups to assess whether changes in myocardial blood flow (MBF) or myocardial flow reserve (MFR) can be demonstrated in relationship with potentially cardiotoxic treatments.

In a recent study comparing MBF and MFR in a small group of women with a history of prior radiation therapy for breast cancer, no difference was found between the anterior and the posterior wall. The authors conclude that there are no direct signs of severe damage in the left anterior descending artery territory, which in theory is more exposed to radiation effects than the inferior wall [38]. Mean MFR values slightly inferior to the normal reference were observed, and since there are no major increases in the coronary artery calcium score, epicardial stenoses are unlikely and a beginning microvascular impairment could be suspected. Nevertheless, this conclusion appears questionable because a major determinant of the reduced MFR was high resting MBF values [38]. Through comparison of rest and stress [^15^O]H_2_O PET pre- versus post-radiation (2 and 8 months) therapy, a decrease in stress MBF in the majority of cases could be assessed, both considering the global values and the left anterior descending segments. Remarkably, none of the patients had a stress MBF below the diagnostic threshold for coronary artery disease [39]. In one study, rest and stress ^82^Rb PET in lymphoma patients before and after the first cycle of doxorubicin therapy were performed. While resting MBF remained unchanged, there was a non-significant decrease in stress MBF and a significantly lower MFR [40]. Furthermore, the authors identified a subgroup of patients with a drop in MFR of more than 20%. The authors speculated that these were patients with a low cardiotoxic threshold, supported only by older age but not by other factors [40].

In conclusion, cardiac perfusion PET may be applied for the effective detection of myocardial ischemia in the context of coronary artery disease in patients candidate to potentially cardiotoxic cancer treatments or as a late sequelae after therapy, to monitor MBF or MFR and cardiotoxic therapy or to explore mechanisms of cardiotoxicity [41].

## Imaging of sympathetic innervation

The current gold standard to evaluate cardiac function in relation to cardiotoxicity due to cancer therapy is the assessment of “systolic” LVEF [2]. However, LVEF will only decrease after a critical mass of myocardial tissue has been damaged [42]. LVEF and early myocardial damage after systemic therapy correlate only weakly, as verified by endomyocardial biopsy [43, 44]. The combination of systemic therapy with radiotherapy may even worsen the burden of cardiotoxicity and has been a topic of extensive research, but it remains a major challenge to identify at-risk patients non-invasively [45]. The compensatory reserve of the myocardium enables sufficient ventricular output, even when structural damage to the myocytes started. Thus, LVEF may underestimate actual cardiac injury [42, 46]. A non-invasive approach such as cardiac innervation imaging is preferable, which accurately identifies cardiotoxicity at a subclinical stage, before decrease in LVEF occurs. Sympathetic nervous innervation imaging of the heart with PET or SPECT is thus a promising tool allowing an evaluation of the disturbed cardiac conducting innervation system. [^123^I]-Metaiodobenzylguanidine ([^123^I]mIBG) is a radiolabeled guanethidine analog, which is taken up, concentrated, and stored in the presynaptic nerve terminals of the sympathetic nervous system in a manner similar to norepinephrine. The role of the dysfunctional autonomic nervous system regulation in heart failure is well established and myocardial sympathetic imaging with [^123^I]mIBG now has a range of proven applications in heart failure patients [47, 48].

Functional and structural injury to myocardial adrenergic neurons may be part of the pathophysiology of cancer therapy-induced cardiotoxicity [49, 50], and assessment of the adrenergic nervous system function of the heart may therefore represent a possible tool for detection of subclinical cardiotoxicity. Despite promising results, [^123^I]mIBG scintigraphy has not yet found its clinical place in the early identification and monitoring of cancer therapy‐induced cardiotoxicity.

Also the role of radiotherapy in the risk of long-term cardiac disease following childhood cancer treatment is evaluated in the association with systemic therapy [51, 52]. One study [52] evaluated the long-term risk of cardiac pathology following radiotherapy and anthracycline for a childhood cancer using [^123^I]mIBG scintigraphy in 447 subjects. This study strongly emphasizes the need to limit heart irradiation during radiotherapy, particularly for patients with adriamycin treatment.

The fate of [^123^I]mIBG imaging in the setting of cardiotoxicity will depend on further investigation in prospective clinical trials, including the application of high sensitive CZT cameras.

Although PET imaging offers the advantages of being even more sensitive and the possibility of absolute quantification, there is as yet no data with established tracers such as [^11^C]metahydroxyephedrine ([^11^C]mHED) or other novel promising PET tracers including the ^18^F-labeled variant [^18^F]flubrobenguane (also known as [^18^F]LMI1195), a novel cardiac neuronal imaging agent with properties similar to [^123^I]mIBG [53], or the ligand CGP12177 for β-receptor density assessment [54].

PET/MR hybrid imaging may be of interest, allowing combined regional molecular and functional LV imaging, that could potentially detect subtle myocardial and molecular signal changes as a very early sign of cardiotoxicity [55].

## Glucose metabolism

PET allows to image changes in cellular metabolism with high sensitivity and appears therefore well suited to identify earlier stages of cardiomyocyte toxicity, before irreversible myocardial damage develops. It must be distinguished here that, on the one hand, cardiotoxic therapy may cause abnormalities in myocardial glucose metabolism, i.e., in the viability assessment of the heart. On the other hand, a myocardial inflammatory reaction may occur as a result of cancer therapy (e.g., immune checkpoint inhibitor-associated myocarditis [56]), which can be sensitively detected by 2-[^18^F]fluoro-2-deoxy-D-glucose (2-[^18^F]FDG) PET when the patient is adequately prepared (i.e., suppression of myocardial glucose metabolism).

Anthracyclines interfere with normal mitochondrial oxidative metabolism causing a shift from lipids to glucose metabolism for energy supply [57]. 2-[^18^F]FDG is taken up by cardiomyocytes through GLUT receptors and then phosphorylated by hexokinase resulting in its intracellular retention. 2-[^18^F]FDG is therefore well suited to identify the metabolic shift in the myocardium in response to anthracycline treatment. Several retrospective studies [58, 59] have found high 2-[^18^F]FDG uptake in the myocardium of patients treated by anthracyclines on PET, which was associated with an increased incidence of LV dysfunction during follow-up. The use of 2-[^18^F]FDG PET for the early identification of cardiotoxicity appears an attractive imaging approach as patients often undergo sequential 2-[^18^F]FDG PET examinations during the course of their oncological disease. However, increased 2-[^18^F]FDG uptake is not specific for anthracycline-induced cardiotoxicity as it is also observed in ischemic myocardium or in patients with elevated circulating insulin levels following sugar consumption. Further carefully conducted studies are required to confirm whether the quantification of myocardial 2-[^18^F]FDG uptake could be a robust early-stage predictor of cardiotoxicity-related LV dysfunction. These studies should be conducted with adequate suppression of cardiac glucose metabolism, as this increases the interpretability of 2-[^18^F]FDG PET/CT studies. Although no standardized guidelines are available, prolonged fasting (beyond 12 h), carbohydrate-restricted diets, fatty meals, and heparin loading have been proposed [60]. A protocol involving high-fat, low-carbohydrate diet on the day prior to scanning followed by prolonged fasting over 12 h is relatively easy to put in place in clinical routine. This can be followed by unfractionated heparin (50 UI/kg) being injected 15 min before the 2-[^18^F]FDG injection. The aim of this protocol is to decrease basal insulin and blood glucose levels and to increase blood free fatty acid (FFA) levels, which shift myocardial energy consumption away from glucose toward FFA. Though the bleeding risk is very low if used properly as a single dose, heparin should be avoided in patients who are already receiving anticoagulant therapy or have a history of bleeding disorders and attention should be paid to tumors at risk of bleeding and brain metastases.

## Assessment of myocardial damage: [^99m^Tc]Tc-annexin/[^111^In]In-antimyosin, fibroblast activation

In addition to the assessment of indirect markers of myocardial damage such as perfusion abnormalities, wall motion abnormalities, or a reduction in LVEF, nuclear medicine techniques are available for the direct assessment of myocardial damage. In this section, radiotracers that target apoptosis or necrosis will be addressed. One approach to visualize myocardial damage is to use the tracer [^111^In]In-antimyosin, a murine monoclonal antimyosin Fab antibody fragment. This tracer visualizes cellular necrosis because it binds to intracellular myosin and only if the sarcolemma is damaged. This tracer has been used in several studies to investigate cancer therapy-induced cardiotoxicity [61, 62, 63, 64]. For example dose-dependent cardiotoxicity of epirubicin was demonstrated [65], and more intense myocardial uptake, in terms of a higher degree of cardiotoxicity, was related to a greater impairment of LV function [65]. However, in another study examining the long-term effects of anthracyclines, both patients who had recovery of LVEF and patients with continued impaired LVEF displayed cardiac tracer accumulation [66]. Accordingly, this study questioned the value of [^111^In]In-antimyosin for prognostic assessment regarding the development of cardiotoxicity-related heart failure. Only limited data are available for imaging radiotherapy-induced cardiotoxicity using this tracer [67].

Phosphatidylserine is a phospholipid that is exposed on the membrane of cells undergoing apoptosis, which can be targeted using [^99m^Tc]Tc-annexin V, a tracer that has not yet been widely used. In a doxorubicin-induced cardiotoxicity model in the rat, the feasibility of imaging with this tracer was demonstrated [68] and the degree of cardiotoxicity in histopathology was related to tracer accumulation [69, 70].

A very recent development in the field is the molecular imaging of activated fibroblasts. Activation of fibroblasts occurs in many cardiac repair and remodeling processes, such as after myocardial infarction, heart failure, and by the administration of cardiotoxic agents. Recently, ^68^ Ga-labeled radiotracers for targeting activated fibroblasts have been developed and have demonstrated extensive fibroblast activation in patients with acute myocardial infarction [71]. Those are quinoline-based radiotracers that function as fibroblast activation protein inhibitors (FAPIs). Initial reports indicate that fibroblast activation as a sign of damage to the myocardium by cardiotoxic agents can be detected using this tracer [72]. However, there is preliminary work indicating that fibroblast activation in the myocardium detected by PET is also associated with pre-existing cardiovascular disease or risk factors and thus is not specific for cancer therapy-induced cardiotoxic injury [73, 74].

## 2-[^18^F]FDG PET for assessment of vascular toxicity

Whereas cancer therapy-related cardiotoxicity remain the prime concern in patients suffering from cancer, the increased risk of vascular disease already posed by the cancer itself is further increased by those therapies. Vascular toxicities are the second most common cause of death in patients with cancer undergoing outpatient therapy [75].

There is a broad range of cancer therapies with a vascular toxicity risk profile, and their effects on the vessel and the related clinical spectrum are quite diverse [75]. Nowadays, sufficient data are available for conventional or targeted chemotherapy-related vascular side effects, respectively. Furthermore, with growing experience, treatment-related thromboembolism, acute vasospasm, and arterial and pulmonary hypertension as well as emerging or progressing atherosclerosis with angina and even acute myocardial infarction and stroke were recognized. It is beyond the scope of this article to illustrate the cardiovascular side effects of each respective chemotherapy protocol. However, almost all of the well-accepted conventional (like alkylating agents, antimetabolites, immunomodulatory drugs) or targeted chemotherapeutics (like proteasome inhibitors, monoclonal antibodies, VEGFR fusion molecules or multitarget kinase inhibitors) carry a relevant individual risk profile for undesired and harmful effects mentioned above [75]. Similarly, although to a lesser degree, radiotherapy-related vascular side effects have been reported, ranging from coronary vasospasm and variant angina in patients suffering from Hodgkin’s lymphoma to adverse effects of radiation on endothelial cell function and viability potentially promoting atherosclerosis [75, 76, 77, 78, 79, 80].

The timeline, in which the vascular sequelae emerge after termination of cancer therapy, is rather heterogeneous and depends on the distinct (pathological) vascular process caused by the oncological treatment. Whereas acute vasospasm frequently emerges within days to weeks and is very likely reversible, development of acute thrombosis frequently lasts weeks to months. Accelerated atherosclerosis as another side effect of cancer treatment has to be expected months to years after the therapy with a very low likelihood of reversibility.

Most of the named vascular side effects of cancer therapies can currently not be assessed by means of nuclear medicine. However, inflammatory changes of the arterial wall in the context of atherosclerosis can reliably be identified by different PET tracers, with by far the most profound experience obtained using 2-[^18^F]FDG. In the beginning of the twenty-first century, the first prospective milestone study in 2-[^18^F]FDG PET imaging of atherosclerosis was published followed by numerous publications on experimental, clinical, and methodological aspects of this topic [81, 82, 83, 84, 85, 86, 87, 88, 89]. Here, not only the correlation between 2-[^18^F]FDG uptake and histologically proven inflammatory changes in arterial specimen, but also with e.g. clinical cardiovascular risk factors as well as with prognostic parameters could be identified [82, 83, 84, 86, 87, 90, 91, 92, 93].

The 2016 position paper of the European Association of Nuclear Medicine on 2-[^18^F]FDG PET imaging of atherosclerosis [94] provides information and recommendations on methodological aspects of this non-invasive imaging approach.

To date, there are limited data on the impact of cancer therapy on vascular inflammation. In a small retrospective study of 10 patients who received radiotherapy for lymphoma, FDG PETs performed 2–7 years after therapy were analyzed. Eighty percent of patients showed higher FDG uptake on the irradiated side compared to the opposite side potentially indicating increased inflammation [95]. In a prospective study of 22 patients with head and neck cancer, FDG PET was performed before and 3 months after radiation therapy. Eighty-two percent of the patients received concurrent chemotherapy. The authors found increased FDG uptake in the carotids after cancer therapy indicating vascular inflammation [96]. In a retrospective study of 52 patients receiving anthracycline-based chemotherapy for Hodgkin lymphoma, FDG PETs were analyzed before and after chemotherapy. None of the arterial segments studied showed increased vascular FDG uptake [97]. There are conflicting data on the effect of ICI therapy on vascular inflammation in humans. While one group described increased arterial FDG uptake in both lymphoma patients [98] and melanoma patients [99] after ICI therapy in small retrospective studies, another group could not reproduce this in their melanoma patients [100]. The latter work also examined the effect of ICI therapy on arterial FDG uptake in an atherosclerotic mouse model. Again, there was no difference in vascular FDG accumulation in these animals, while at the same time, a marked increase in cytotoxic CD8^+^ T cells was detected in the inflammatory plaques after ICI therapy and accelerated atherosclerosis was described.

In summary, there are currently limited data on the detection of cardiotoxic effects on vessels after cancer therapy and further studies are needed to assess the utility of this imaging modality.

## Future perspectives

### Promising imaging approaches for future studies

As outlined in the previous sections, there are already several imaging approaches to detect cardiotoxic cardiovascular disease. Apart from those already clinically evaluated, there are other sets of radiotracers with great potential for the detection of cardiac damage caused by cardiotoxic therapies (see Table 6). In addition to the ^68^Ga-labeled FAPI tracer mentioned above for the detection of activated fibroblasts as a sign of cardiac damage [72], an ^18^F-labeled tracer for imaging sympathetic innervation is available ([^18^F]F-flubrobenguane) [101], the use of which is expected to be approved in the near future. However, data on the detection of cardiotoxicity are still lacking. Furthermore, there are promising tracers for imaging activated macrophages (e.g., ^68^Ga-labeled somatostatin receptor agonists) [102, 103] and for imaging neovascularization, with the most common tracer targeting the integrin α_V_β_3_ [104]. Most recently, initial preclinical work attempted to use radiotracers for imaging cardiotoxicity via detection of mitochondrial damage [105, 106] or reactive oxygen species (ROS) formation [107]. Research projects assessing these promising tracers are urgently warranted.

**Table 6:** Potential targets and promising radiotracers for cardio-oncology in the future

Target	Involved processes	Tracers	Potential applications	Ref
Somatostatin receptors (SSTR)	Overexpression on macrophages	[^68^Ga]Ga-DOTATOC, [^68^Ga]Ga-DOTATATE, [^68^Ga]Ga-DOTANOC	Inflammatory processes (e.g., myocarditis, pericarditis, vasculitis)	[102, 103]
α_v_β_3_ integrin receptor	Cell adhesion, neoangiogenesis, overexpressed on macrophages	[^18^F]F-galacto-RGD, [^68^Ga]Ga-PRGD2, [^18^F]F-fluciclatide	Neoangiogenesis, inflammatory processes	[104]
Fibroblast activation protein (FAP)	Activation of fibroblasts	Various ^68^Ga-labeled inhibitors of FAP (e.g., [^68^Ga]Ga-FAPI-04, [^68^Ga]Ga-FAPI-46)	Myocardial damage	[73]
Norepinephrine transporter (NET)	Sympathetic innervation of the heart	[^18^F]F-flubrobenguane	Denervation as early sign of damage to the heart	[101]
Mitochondrial membrane potential	Dysfunction of mitochondrial membrane	[^18^F]F-MitoPhos, [^68^Ga]Ga-Galmydar	Mitochondrial dysfunction as early sign of cardiotoxicity	[105, 106]
Reactive oxygen species (ROS)	Superoxide production in processes such as myocardial apoptosis or necrosis	[^18^F]F-DHMT	ROS generation as early sign of cardiotoxicity	(107)

### Approach for nuclear imaging assessment in the field of cardiotoxicity

**Fig. 4:**
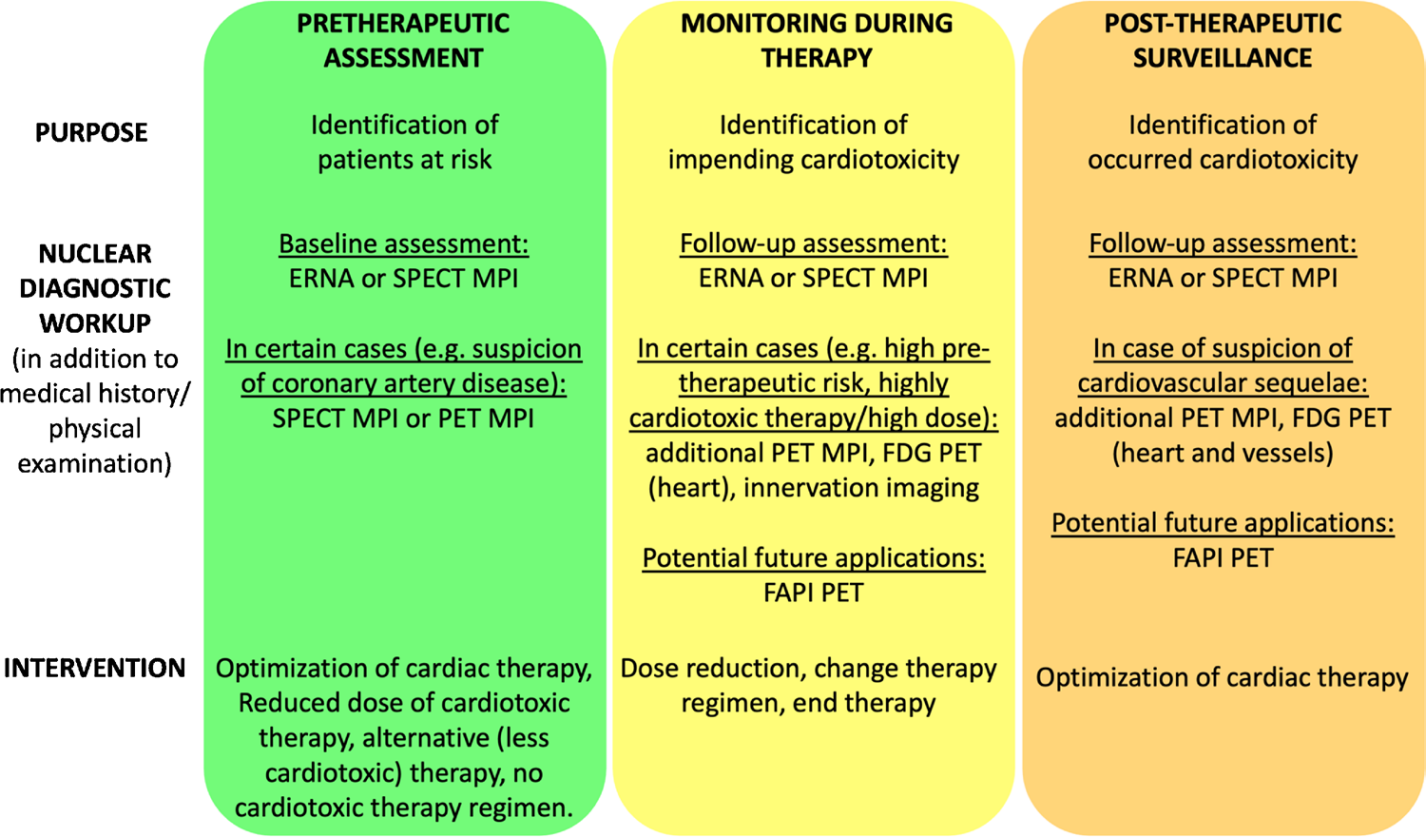
Suggested approach to nuclear medicine imaging in the field of cardiotoxicity

Figure 4 shows a suggested approach to nuclear medicine imaging in the field of cardiotoxicity and with regard to normal values, we recommend referring to Tables 2 and 5, respectively. From the armamentarium of nuclear imaging, ERNA or SPECT MPI are particularly suitable for the investigation of pump function. If obstructive coronary artery disease is suspected, it can be evaluated by SPECT or PET MPI. This pretherapeutic assessment is used to adjust the dose of the planned therapy in case of pre-existing cardiac disease, to consider an alternative therapy regimen, or to refrain from cardiotoxic therapy completely. Furthermore, ERNA and SPECT MPI are alternative methods if echocardiography or CMR are not feasible or available. After initiation of therapy, regular assessment is necessary to detect cardiotoxicity at an early stage. In addition to reassessment of pump function by ERNA or SPECT MPI, PET MPI is particularly useful in cases of suspected microvascular disease and a new onset of this condition or a significant decrease in MBF or MFR with cancer therapy is indicative of cardiotoxicity and should lead to modification of therapy. 2-[^18^F]FDG PET is useful to detect altered metabolism of the myocardium or to detect cardiac inflammatory effects. Imaging of damage of myocardial innervation may also be considered as an early sign of cardiotoxicity. In particular, [^68^Ga]Ga-FAPI PET appears to be a potential promising application for the future, although this remains to be evaluated in studies. These very sensitive methods should be used when CMR, echocardiography, ERNA, and SPECT show no abnormalities or unclear findings and cardiotoxicity is still suspected. If PET MPI shows a decrease in MBF or MFR, or a new onset of microvascular dysfunction, this is a sign of cardiotoxicity and should lead to a modification of the therapeutic regimen. Similarly, a new abnormality on FDG PET of the heart should lead to a modification of the therapeutic regimen (dose reduction, change of therapeutic regimen, or discontinuation of therapy). After completion of therapy, reassessment of cardiac function by ERNA or SPECT MPI is reasonable to detect myocardial damage that has occurred if echocardiography or CMR are not feasible or available. Furthermore, the imaging method selected before the initiation of therapy should be chosen for follow-up for better comparability.

PET MPI and 2-[^18^F]FDG PET also seem to be useful as further diagnostics. 2-[^18^F]FDG PET can also be used to determine whether increased inflammatory activity in the vessels has occurred as a result of cardiotoxic therapy; however, this represents a future application, as only little data is available and the value should be further investigated. [^68^Ga]Ga-FAPI PET appears to be a promising future imaging modality to detect cardiotoxic cardiac injury at a very early stage. Also [^68^Ga]Ga-FAPI PET allows to distinguish between active fibrosis and mature scars. Therefore, [^68^Ga]Ga-FAPI PET can be considered when conventional imaging modalities have been unremarkable or non-conclusive and cardiotoxicity remains suspected. If cardiotoxic injury is detected, further cardiologic evaluation and initiation of therapy should follow.

## Importance of interdisciplinary cooperation

The topic of cardiotoxicity is complex. The diagnosis and therapy of a cardiovascular disease due to a previous cancer therapy therefore requires a close interdisciplinary approach including cardiologists, oncologists, radiation therapists, and imaging disciplines (radiology and nuclear medicine), among others. Only this interplay of disciplines will provide optimal care for cancer patients or cancer survivors and prevent secondary diseases of the heart. This is also the reason why centers are creating innovative units as an interface between different specialties to meet this increased need for interdisciplinarity. One example of this is the clinical unit of “Nuclear Cardiology” at the University Hospital Essen [108]. Last but not least, we agree with the recommendation of international societies that the formation of the so-called cardio-oncology teams is an important pre-requisite to monitor not only current tumor patients but also recovered patients with the risk of cardiovascular complications [2]. Such cardio-oncology teams are also necessary for early detection of cardiovascular complications of novel therapies.

## Conclusion

Cardio-oncology represents an important new field that should be covered by multiple specialties as part of interdisciplinary teams. Nuclear medicine can provide important insights in the early detection of impending cardiotoxicity, assist in the monitoring of cardiotoxic therapy, and may also be used as a tracking tool in the investigation of cardiotoxicity of novel therapies. While nuclear cardiology has many promising techniques available, some of which are already being used in routine clinical practice, further studies are needed to investigate the full value of nuclear medicine techniques in the care of patients treated with cardiotoxic agents.

## Liability statement

This article summarizes the views of the EANM Cardiovascular Committee and the EANM Oncology & Theranostics Committee. It reflects recommendations for which the EANM cannot be held responsible. The recommendations should be taken into context of good practice of nuclear medicine and do not substitute for national and international legal or regulatory provisions.
