# Correction to: Nuclear medicine in the assessment and prevention of cancer therapy‑related cardiotoxicity: prospects and proposal of use by the European Association of Nuclear Medicine (EANM)

**DOI:** 10.1007/s00259-022-06039-6

**Published:** 2022-11-21

**Authors:** Matthias Totzeck, Nicolas Aide, Johann Bauersachs, Jan Bucerius, Panagiotis Georgoulias, Ken Herrmann, Fabien Hyafil, Jolanta Kunikowska, Mark Lubberink, Carmela Nappi, Tienush Rassaf, Antti Saraste, Roberto Sciagra, Riemer H.J.A. Slart, Hein Verberne, Christoph Rischpler

**Affiliations:** 1 Department of Cardiology and Vascular Medicine, West German Heart and Vascular Center Essen, University Hospital Essen, University Duisburg-Essen, Essen, Germany; 2 Nuclear Medicine Department, University Hospital, Caen, France; 3 Department of Cardiology and Angiology, Hannover Medical School, Hannover, Germany; 4 Department of Nuclear Medicine, University Medicine Göttingen, Georg-August-University Göttingen, Göttingen, Germany; 5 Department of Nuclear Medicine, Faculty of Medicine, University of Thessaly, University Hospital of Larissa, Larissa, Greece; 6 Clinic for Nuclear Medicine, University Hospital Essen, University of Duisburg-Essen, Essen, Germany; 7 Department of Nuclear Medicine, DMU IMAGINA, Georges-Pompidou European Hospital, Assistance-Publique – Hôpitaux de Paris, University of Paris, Paris, France; 8 Nuclear Medicine Department, Medical University of Warsaw, Warsaw, Poland; 9 Medical Physics, Uppsala University Hospital, Uppsala, Sweden; 10 Department of Advanced Biomedical Sciences, University of Naples “Federico II”, Naples, Italy; 11 Heart Center, Turku University Hospital and University of Turku, Turku, Finland; 12 Nuclear Medicine Unit, Department of Experimental and Clinical Biomedical Sciences “Mario Serio”, University of Florence, Florence, Italy; 13 Medical Imaging Center, Department of Nuclear Medicine and Molecular Imaging, University of Groningen, University Medical Center Groningen, Groningen, The Netherlands; 14 Department of Biomedical Photonic Imaging, Faculty of Science and Technology, Enschede, The Netherlands; 15 Department of Radiology and Nuclear Medicine, Amsterdam UMC, Location AMC, University of Amsterdam, Amsterdam, The Netherlands

**Correction to** **:** **European Journal of Nuclear Medicine and Molecular Imaging**

https://doi.org/10.1007/s00259-022-05991-7

The authors regret that their names in the original article are incorrect as the first and last names were interchanged. It is now corrected in this erratum article.

The original article can be found at https://doi.org/10.1007/s00259-022-05991-7.

